# Psychische Gesundheit und Geschlecht in der zweiten Hälfte des 20. Jahrhunderts. Einleitung: Verflochtene Geschichte von psychischer Gesundheit und Geschlecht: Forschungsstand und offene Fragen

**DOI:** 10.1007/s00048-024-00409-x

**Published:** 2024-12-04

**Authors:** Ulrike Klöppel, Susanne Doetz, Vera Luckgei

**Affiliations:** 1 Institut für Geschichte und Ethik der Medizin, Ruprecht-Karls-Universität Heidelberg, Im Neuenheimer Feld 327, 69120 Heidelberg, Deutschland; 2 Institut für Geschichte der Medizin und Ethik in der Medizin, Charité – Universitätsmedizin Berlin, Thielallee 71, 14195 Berlin, Deutschland

In den 1970er Jahren empörten sich Feministinnen darüber, dass Frauen, „um in den Augen der Gesellschaft als gesund zu gelten, Verhaltensweisen übernehmen [müssen], die gesellschaftlich geringer bewertet und in Relation zu männlichen Verhaltensweisen als krank bezeichnet werden“ (Burgard 1977: 67). Die feministische Kritik galt sowohl den Weiblichkeitsnormen als auch ihrer Verflechtung mit Verhaltensbewertungen als „normal“ oder „pathologisch“. Änderten sich mit der feministischen Kritik an den Geschlechternormen allmählich auch die Normen psychischer Gesundheit? Historisch lässt sich ihr Nexus bis mindestens ins 19. Jahrhundert zurückverfolgen. In der *Encyklopädie der gesammten medicinischen und chirurgischen Praxis* von 1833 ließ sich unter dem Lemma „Constitutio muliebris“ nachlesen:

Das Weib hat, um seiner Bestimmung als Gattin, Mutter und Hausfrau zu entsprechen, einen ganz eigenthümlichen Charakter. Es drückt sich aus durch grosse Zartheit und Reizbarkeit des Körpers, durch die Verschiedenheit der Sexualorgane und deren Einfluss auf Geist und Körper (Most 1833: 468).

Das „reizbarere Nervensystem“ (ebd.), so heißt es weiter, mache das weibliche Geschlecht zum krankheitsanfälligeren. Wie Claudia Honegger (1996) dargelegt hat, prägten die Medizin, Anthropologie und Philosophie im Kontext des sozialen Aufstiegs des Bürgertums ein dichotomes Verständnis von Geschlecht. Diese Konstruktion wurde unter anderem durch den Dualismus von psychischer Gesundheit und Krankheit untermauert, wobei die Frau qua ihrer Konstitution am Rande des Nervenzusammenbruchs verortet wurde.

In der Forschungsliteratur ist die Verschränkung der Kategorien Geschlecht und normal/verrückt unter anderem am Beispiel psychiatrischer Diagnosen,^1^ der Begründung von Devianz in medizinischen Gutachten (u. a. Carrier 2017; Herrn 2014; Bock 2010), an der Werbung für und der Behandlung mit Psychopharmaka (Antidepressiva, Hirshbein 2006; Tranquillizer, Tone 2009) sowie an der Repräsentation der „wahnsinnigen“ Frau in Kunst und Literatur untersucht worden (u. a. Showalter 1985; Schlichter 2000).^2^ Auch wurde gezeigt, dass der für die psychiatrische Einweisung so zentrale Vorgang der Entmündigung bürgerlicher Männer als Verweiblichung gewertet wurde (Bernet 2007; Goldberg 2003). Der Großteil der bisherigen historischen Forschung bezieht sich allerdings auf das 19. und die erste Hälfte des 20. Jahrhunderts. Wie – so fragen wir mit dem vorliegenden Themenheft – veränderte sich in der zweiten Hälfte des 20. Jahrhunderts unter dem Druck der Neuen Sozialen Bewegungen und eines antibürgerlichen, linksalternativen Milieus die Co-Konstruktion von Geschlechter- und psychischen Normen?^3^

Die wenigen zeitgeschichtlichen Studien zur zweiten Hälfte des 20. Jahrhunderts, die den Nexus von Geschlecht und psychischer Gesundheit adressieren, haben vor allem Kontinuitäten der „bürgerlichen“ Differenzkonstruktionen herausgearbeitet (Metzl 2002; Hirshbein 2006; Weinstein 2018).^4^ Für die USA, Kanada und England haben historische Untersuchungen rekonstruiert, wie die feministische Bewegung beginnend mit den 1970er Jahren Einfluss auf die (akademische) Psychologie (Rutherford & Granek 2010; Pickren & Rutherford 2010; Johnston & Johnson 2017), auf die psychologischen Berufsvertretungen (Pyke 2001; Chrisler et al. 2013), auf die psychotherapeutische Praxis (Brodsky 1973; Kim & Rutherford 2015; Ruck 2015; Brown 2018; Mahoney 2023) sowie auf Praktiker*innen (Pitts & Kawahara 2018) nahm und deren stereotype Weiblichkeitszuschreibungen infrage stellte. Ähnliches haben aktuelle Untersuchungen zur therapeutischen Frauenberatung (Ruck et al. 2022) und der universitären Lehre frauenspezifischer Psychologie (Luckgei et al. 2020) seit den 1980er Jahren in Wien gezeigt. Welchen Einfluss der Protest der Neuen Frauenbewegung in Westdeutschland gegen Frauenfeindlichkeit, patriarchale Dominanz, Ignoranz und Doppelstandards im psychiatrischen, psychologischen und therapeutischen Feld auf die gesellschaftliche Wahrnehmung von Geschlecht ausübte, ist hingegen bislang unerforscht. Auch die (De‑)Konstruktion von Männlichkeit ist für die zweite Hälfte des 20. Jahrhunderts in der psychiatriehistorischen Literatur noch kaum untersucht worden. Lediglich Christoph Schwamm (2018) stellt Männlichkeit in den Mittelpunkt seiner Untersuchung, bleibt jedoch auf die psychiatrische Anstalt konzentriert.^5^ Historische Arbeiten über den in der Bundesrepublik der 1970er Jahre einsetzenden Trend zur Selbstnormalisierung und -optimierung durch psychologische, sozialpädagogische, therapeutische, antipsychiatrische und psychosoziale Beratungsangebote nehmen wiederum bis auf wenige Ausnahmen kaum Bezug auf Verschränkungen mit Geschlechtskonstruktionen.^6^ Dasselbe gilt für Studien zur westdeutschen Psychiatriereform sowie zu psychiatriekritischen und antipsychiatrischen Initiativen, welche die psychiatrische Disziplin als solche infrage stellten oder einzelne ihrer Aspekte wie Anstaltsverwahrung, Elektroschocks, medikamentöse Ruhigstellung oder psychopathologisierendes Labeln anprangerten.^7^

## Transformationsprozesse in der zweiten Hälfte des 20. Jahrhunderts: Die Beiträge

Das vorliegende Themenheft richtet den Fokus auf die zum Teil ungleichzeitigen Diversifizierungsprozesse der verschränkten Konstruktionen von Geschlecht und psychischer Gesundheit/Alterität im psychosozialen Feld der Bundesrepublik seit den 1950er Jahren. Diese waren durch eine zunehmende Psychologisierung, Pädagogisierung und Therapeutisierung geprägt und erstreckten sich auch in den ambulanten Bereich. In diesem Psy-Sektor (Klöppel im Erscheinen) entstanden im Kontext der Frauenbewegung neue Ansätze, die das vorherrschende Geschlechterverständnis infrage stellten und die patriarchale Lebensrealität von Frauen berücksichtigten. In welcher Weise trugen sie, indem sie das herkömmliche Frauenbild kritisierten, zu Veränderungen der dualistischen Wahrnehmung von psychischer Gesundheit und Krankheit bei? Und beförderten solche Ansätze auch einen Wandel der psychosozialen, psychologischen und psychiatrischen Versorgungslandschaft? Die Beiträge des Themenhefts verdeutlichen Wandlungsprozesse, aber auch gegenläufige Tendenzen in Form antiemanzipativer und rebiologisierender Beharrungskräfte und Abwehrkämpfe.

Wie **Vera Luckgei **in ihrem Beitrag auseinandersetzt, traten in der Bundesrepublik anfänglich vor allem Psychologinnen mit einer Kritik am Genderbias des psychologischen Diskurses und der psychotherapeutischen Praxis in Erscheinung. Sie gründeten eine Berufsvertretung für Psychologinnen, setzten sich für Frauenforschung in der Psychologie ein und formulierten feministische Prinzipien für emanzipative therapeutische Angebote von Frauen für Frauen. Auf die damals noch weitgehend männlich dominierte akademische Psychologie vermochten sie keinen prägenden Einfluss auszuüben. Dagegen verlagerten viele Psychologinnen in den späten 1970er und 1980er Jahren ihr Engagement in den Bereich der Praxis und wirkten an der Entstehung des feministischen Psy-Sektors mit, indem sie eine Vielzahl von Angeboten und Projekten feministischer Therapie, Beratung und sozialpädagogischer Prävention schufen. **Susanne Doetz** macht am Beispiel der feministischen Zeitschrift *Courage* deutlich, wie die Praktiken rund um deren Herstellung seit Ende der 1970er Jahre die Entstehung eines feministischen Gegenwissens zur herkömmlichen Psychiatrie und Therapie beförderten. Die Zeitschrift bot ein experimentelles Forum, in dem die Erfahrungen von Frauen mit Psychiatrie und Therapie mit einer feministischen Kritik an der überproportionalen Psychopathologisierung von Frauen und patriarchalen Machtstrukturen verknüpft wurden. Die Analysen von Luckgei und Doetz verdeutlichen, wie sich ein feministischer Psy-Sektor im Sinne eines alternativen Praxis- und Wissensfeldes entwickelte, das sich kritisch von den Strukturen der traditionellen psychosozialen und psychiatrischen Versorgung sowie teilweise vom alternativen Psychomarkt abgrenzte. Auch der Beitrag von **Ronda Ramm, Beate Binder** und **Francis Seeck **nimmt Praktiken in den Blick, die sich kritisch auf die Psychiatrie bezogen. Sie untersuchen Politiken der provokanten und in Stolz umgewendeten öffentlichen Sichtbarmachung von psychischer Alterität anhand der psychiatriekritischen öffentlichen Umzüge der „Blauen Karawane“ (ab den 1980er Jahren) sowie den Paraden „behindert und verrückt feiern“ (ab 2013). Während beide öffentlichen Interventionen darauf abzielten, die Grenzziehung zwischen normal und verrückt sowie die gesellschaftliche Abwertung zu überwinden, nahm die Parade „behindert und verrückt feiern“ darüber hinaus psychiatrische Praktiken der Perpetuierung von Heteronormativität aufs Korn.

Zwei weitere Beiträge des Themenheftes beschäftigen sich mit den Entwicklungen der etablierten Fachdiskurse zu Alkoholsucht von Frauen und zu „psychisch kranken Straftäterinnen“ in der Bundesrepublik. **Viola Balz** zeigt anhand der in Westdeutschland in den 1950er Jahren einsetzenden psychiatrischen, psychologischen und pädagogischen Diskussion über zunehmenden Frauenalkoholismus, wie dieser als Nebeneffekt feministischer Emanzipationsbestrebungen gedeutet wurde. Zugleich galt er auch als Ausdruck des allgemeinen „Wohlstandsalkoholismus“. Diese Deutungen brachten mit der Hervorhebung der gesellschaftlichen Bedingtheit eine Abkehr vom psychiatrischen Paradigma der erbbiologischen Erklärung mit sich – verbunden mit einer Stärkung therapeutischer, pädagogischer und psychologischer Hilfsangebote. Diese Neuausrichtung des Feldes wurde verstärkt durch feministische Interventionen, Forschungsansätze und Projekte für süchtige Frauen, die im Alkoholismus eine selbstzerstörerische Reaktion von Frauen auf das Leiden unter patriarchaler Einengung und Gewalt sahen. **Chantal Marazia, Uta Hinz **und **Heiner Fangerau** zeichnen den juristisch-kriminologischen sowie den forensisch-psychiatrischen Diskurs am Schnittpunkt zwischen weiblicher Krankheit, Gefährlichkeit und Kriminalität nach. Im Kontext sich wandelnder Gesellschafts- und Rollenbilder wiesen seit den späten 1960er Jahren Wissenschaftlerinnen auf die verbreitete Ignoranz gegenüber sozialisationsbedingten Formen weiblicher Straftaten in Kriminologie, Forensik und an Gerichten sowie auf die Persistenz biologistischer, stereotypisierender Geschlechtszuschreibungen hin. Doch blieben solche kritischen Einwände Randerscheinungen und fanden bis in die 1990er Jahre kaum Eingang in forensisch-psychiatrische Erörterungen zu Fragen der Schuldfähigkeit und Gefährlichkeitsprognose.

Zusammengenommen zeigen die Beiträge, wie und auf welch unterschiedliche Weise die Frauenbewegung innerhalb des weit gefassten Psy-Sektors auf das Verständnis von Geschlecht und damit zugleich auch auf die Wahrnehmung von psychisch krank/gesund wirkte. In den untersuchten Bereichen des Psy-Sektors entfaltete sich feministisches Gedankengut auf unterschiedliche Weisen und mit großen Differenzen hinsichtlich der Bedeutung für den jeweiligen Gegenstand. Feministische Akteurinnen und deren autonome Organisationsstrukturen, aber auch die Produktion und Verbreitung feministischen Gegenwissens, die im Zentrum der Beiträge von **Luckgei** und **Doetz** stehen, bauten eher von außen Druck auf den etablierten Psy-Sektor auf. Dagegen beleuchten **Balz** und **Marazia et al.**, wie sich feministische Impulse innerhalb der wissenschaftlichen Debatte rund um Alkoholkonsum und in der Forensik artikulierten. Während sie in der Forensik allerdings kaum Wirkungen entfalten konnten, führte die feministische Sichtweise auf den Alkoholkonsum von Frauen zu neuartigen Ansätzen in der Prävention und Forschung. Die Integration feministischer Ansätze und Projekte in den Psy-Sektor öffnete allmählich das Feld für ein diversifiziertes Geschlechterverständnis. Dadurch wurden das Aufwachsen in unterschiedlichen Lebensrealitäten und insbesondere die psychischen Folgen von Gewalt gegen Mädchen und Frauen berücksichtigt. Vergeschlechtlichte Krankheitszuschreibungen schwanden allmählich. **Ramm et al.** fokussieren den psychiatriekritischen Aktivismus, der seit der Jahrtausendwende vermehrt queer-feministische Positionen einbindet und damit auch Akteur*innen sichtbar macht, die intersektionale Diskriminierungen im Psy-Sektor und in der gesamten Gesellschaft anprangern. Die Paraden machen einen positiven Zugang zur Vielfalt von Geschlechtern, Sexualitäten, Körpern und psychischen Zuständen als heterotope Momente erfahrbar.

Während also feministische, später auch queere Positionen an einigen Stellen den Psy-Sektor nachhaltig herausforderten, wurden sie in anderen Bereichen als vermeintliche Störfaktoren weitgehend ausgeklammert und an den Rand gedrängt. Das Zusammenspiel der Beiträge macht einerseits unterschiedliche Facetten eines Wandels sichtbar und zugleich die Veränderungsresistenz des Psy-Sektors – so etwa die Kontinuitäten der Marginalisierung mehrfach stigmatisierter Frauen im Beitrag von **Marazia et al.** Letztendlich muss die Etablierung des feministischen Psy-Sektors im Kontext der Psychiatriereform der 1970er und 1980er Jahre betrachtet werden. Diese stärkte den ambulanten Bereich und eröffnete damit insbesondere Sozialarbeiter*innen und Psycholog*innen – darunter zunehmend Frauen – neue Tätigkeitsfelder. Vor allem die im Psy-Sektor Beschäftigten können als Gewinner*innen der Reformbestrebungen gelten, außerdem jene, die in der Lage waren, die neuen Angebote erfolgreich für sich zu nutzen (Reumschüssel-Wienert 2021: 172 ff.). Daraus abgeleitet ließe sich konstatieren, dass die Wirkung der Frauenbewegung eher in einer Erweiterung des Psy-Sektors bestand als in der Abschaffung psychiatrischer Einrichtungen. Dieser Befund würde unter anderem die weitgehende Unverbundenheit von Frauenbewegung und psychiatriekritischem Aktivismus in den 1980er Jahren erklären.

Die Beiträge verdeutlichen, wie über feministische Kritik unterschiedliche Bereiche des Psy-Sektors miteinander verwoben wurden. Darin sehen wir eine der Stärken des vorliegenden Themenheftes. Für Bereiche, in denen feministische Einflüsse weniger präsent waren, bieten jene Beiträge, in denen die Debatten und Praktiken der Frauenbewegung im Zentrum stehen, Kontraste und Kontextualisierungen. Für Bereiche, in denen feministische Positionierungen dominieren, erlaubt der Blick in andere Felder wie die Forensik, die Grenzen des realen Einflusses innerhalb von Wissenschaft und gefestigten Institutionen nachzuvollziehen. Zusammengenommen tragen die Beiträge zu einer geschlechterhistorischen Differenzierung der zeitgeschichtlichen Forschung zum psychosozialen, psychiatrischen und therapeutischen Feld bei, indem sie ungleichzeitige und zum Teil gegenläufige Entwicklungen sichtbar machen. Zugleich liefern sie Impulse für die geschlechtergeschichtliche Forschung, sich mit der Entwicklung von Praktiken und Wissensbeständen dieses Feldes nach 1945 stärker auseinanderzusetzen.

## Footnotes

1 Zur Hysterie siehe u. a. Fischer-Homberger (1969); Schaps (1992); Nolte (2003); zur Neurasthenie vgl. u. a. Radkau (1998); Kaufmann (2001); Forth (2001); Schmiedebach (2001); Hofer (2021). Für aus der Zweiten Frauenbewegung heraus formulierte kritische Beschäftigungen mit der Reproduktion patriarchaler Geschlechterverhältnisse durch Psychiatrie und Psychoanalyse vgl. u. a. Chesler (1997) [1972]; Burgard (1977).

2 Schlichter (2000) hat aus einer literaturwissenschaftlichen Perspektive die „Figur der verrückten Frau“ kritisch analysiert, der sie eine konstitutive Funktion für die feministische Psychiatriekritik und Theoriebildung zuschreibt. Sie problematisiert die feministische Aneignung und Universalisierung der Figur als Prototyp weiblicher Unterdrückung, insofern diese die Analogie von Weiblichkeit und Wahnsinn reproduziere. Für die Theoriebildung der Frauenbewegung in Westdeutschland war auch die kritische Auseinandersetzung mit der Psychoanalyse prägend, wie Katharina Lux (2022) in ihrer Untersuchung der feministischen Zeitschrift *Die Schwarze Botin* zeigt.

3 Das Themenheft ist aus dem Workshop „Psychische Gesundheit und Geschlecht in der zweiten Hälfte des 20. Jahrhunderts“ hervorgegangen, der vom Institut für Geschichte und Ethik der Medizin der Universität Heidelberg am 19. und 20.10.2023 ausgerichtet wurde. Der Workshop fand im Rahmen der DFG-geförderten Forschungsgruppe „Normal#Verrückt. Zur Erosion einer erodierenden Differenz“ statt.

4 Wir können hier nur exemplarisch auf Studien, die die für unser Thema relevanten Kontexte der Bundesrepublik eruieren, hinweisen. Zur Geschlechtergeschichte, insbesondere der Neuen Frauenbewegung, vgl. z. B. Lenz (2008); Paulus et al. (2012); Heinemann & Steber (2021). Zur Familiengeschichte siehe u. a. Neumaier (2019); Limper (2021). Zur Psychiatrie‑, Psychologie- und Psychotherapiegeschichte vgl. u. a. Maasen et al. (2011); Wieser & Malich (2021).

5 In anderen historischen Studien ist psychisches Leid von Männern zwar explizit Thema (z. B. Goltermann 2009), jedoch nutzen diese Gender nicht als Analysekategorie.

6 Für die USA vgl. Castel et al. (1982); für den deutschsprachigen Raum siehe Maasen (2011); Tändler (2016); Eitler & Elberfeld (2015); Elberfeld (2020). Ausnahmen sind u. a. die Studie von Wellmann (2011) zu Sexualtherapien, die Veränderungen heterosexueller Paarbeziehungen als Ziel und Effekt der therapeutischen Praxis herausarbeitet. Ruck et al. (2020) zeigen am Beispiel der psychologischen Frauenberatung, wie vergeschlechtlichte Arbeitsteilung und psychosoziale Beratungspraxis miteinander verschränkt sind. Klöppel (im Erscheinen) geht dem Subjekt- und Politikverständnis feministisch-therapeutischer Ansätze in der Bundesrepublik der 1970er und 1980er Jahre nach. Ihre Studie zeigt, dass sich feministische Therapie und Beratung vom Selbstverwirklichungsideal des Psychobooms distanzierte; psychische Gesundheit von Frauen sollte ausgehend von solidarischen Beziehung zu anderen Frauen aufgebaut werden. Topal-Cevahir (2018) thematisiert in ihrer Oral-History Studie über die Gesundheitsverhältnisse türkischer Migrantinnen die Verschränkung von Migration, (psychischer) Gesundheit und Gender und arbeitet die Stigmatisierungen und Stereotypisierungen, mit denen diese konfrontiert sind, heraus.

7 Siehe u. a. Kersting (2003); Brink (2010); Pross (2016); zur Nieden & Korecky (2018); Hemmerling (2019); Söhner (2020); Reumschüssel-Wienert (2021); Dörre (2021); Beyer (2022). Gerhards (2022) zieht zwar in ihren Ausführungen zur Genealogie eines kollektiven Patientensubjekts am Beispiel des Sozialistischen Patientenkollektivs (SPK) Parallelen zur Frauenbewegung, lässt aber Genderaspekte in Bezug auf das SPK selbst unberücksichtigt.
